# Mitophagy in the mechanisms of treatment resistance in solid tumors

**DOI:** 10.3389/or.2025.1607983

**Published:** 2025-07-21

**Authors:** Xiaoyi Yan, Hui Ding, Mengxiao Ren, Lei Zang

**Affiliations:** The Third Department of Geriatrics, Weifang People’s Hospital, Weifang, Shandong, China

**Keywords:** mitophagy, solid tumors, treatment resistance, cellular autophagy, tumor biology

## Abstract

This review aims to explore the mechanisms by which mitophagy contributes to treatment resistance in solid tumors. As advancements in cancer therapies continue to evolve, treatment resistance emerges as a significant barrier to successful tumor management. Mitophagy, a specific form of cellular autophagy, has been implicated in the survival, proliferation, and drug resistance of tumor cells. This article will summarize the latest research findings and analyze how mitophagy impacts the biological characteristics of solid tumors, thereby revealing its potential implications in cancer treatment strategies. By understanding the role of mitophagy in the context of treatment resistance, we may uncover new therapeutic targets and strategies to enhance the efficacy of existing cancer treatments.

## 1 Introduction

Mitophagy is a specialized form of autophagy that serves to maintain mitochondrial quality by removing dysfunctional mitochondria, thus ensuring cellular homeostasis (1), which is regulated by several key proteins, including PTEN-induced kinase 1(PINK1) and Parkin (2). Research indicates that cancer cells often exploit mitophagy to adapt to the metabolic demands imposed by therapeutic interventions. For instance, studies have shown that enhanced mitophagy can confer resistance to chemotherapeutic agents by enabling cancer cells to survive the oxidative stress induced by these drugs (3, 4). This protective mechanism allows tumor cells to maintain their bioenergetic status and resist apoptosis, ultimately leading to treatment failure. Moreover, the tumor microenvironment (TME) plays a significant role in modulating mitophagy and, consequently, treatment responses. And the interplay between mitophagy and other cellular pathways, such as those governing inflammation and immune evasion, can further complicate the therapeutic landscape. For example, the activation of mitophagy has been linked to the suppression of immune responses.

Emerging evidence suggests that targeting mitophagy could represent a novel therapeutic strategy to overcome treatment resistance in solid tumors. For instance, the inhibition of mitophagy has been associated with increased apoptosis in cancer cells exposed to doxorubicin, a common chemotherapeutic agent (5). Despite these advances, critical knowledge gaps persist, such as the dual roles of mitophagy in treatment responses, which remain unclear. No isoform-specific inhibitors exist for cancer-associated mitophagy targets in the current clinic. And microenvironment crosstalk may interfere with mitophagy-target therapies. These gaps highlight a great need for a systematic review to summarize the relationship between mitophagy and treatment resistance.

Our review aims to synthesize current knowledge on the mechanisms by which mitophagy contributes to treatment resistance, explore potential therapeutic interventions, and discuss future directions for research in this critical area of oncology.

## 2 Results

### 2.1 Definition and key regulators of mitophagy

Mitophagyis a selective degradation process that targets dysfunctional or superfluous mitochondria for lysosomal degradation (1). Once the mitochondria are marked, they are engulfed by autophagosomes, which then fuse with lysosomes to form mitolysosomes. The regulation of mitophagy involves a complex network of signaling pathways and molecular players. PINK1 and Parkin are the most studied components, forming a central axis in the mitophagy pathway. PINK1 acts as a sensor of mitochondrial health, accumulating on depolarized mitochondria and activating Parkin, which ubiquitinates target proteins on the outer mitochondrial membrane, marking them for degradation (2, 6, 7). In addition to PINK1 and Parkin, other proteins such as BCL2/adenovirus E1B 19 kDa protein interacting protein 3 (BNIP3) (8) and FUN14 domain containing 1 (FUNDC1) (9)^(p1)^ also play critical roles as mitophagy receptors that facilitate the recruitment of autophagic machinery to damaged mitochondria.

Moreover, the interplay between mitophagy and various cellular signaling pathways is crucial for its regulation. For instance, the AMP-activated protein kinase (AMPK) pathway is activated in response to energy stress and can stimulate mitophagy by promoting the expression of autophagy-related genes. The mTOR pathway inhibits autophagy under nutrient-rich conditions, thereby preventing unnecessary degradation of cellular components (10). In addition to these pathways, post-translational modifications such as phosphorylation and ubiquitination are essential for the regulation of mitophagy. For example, the phosphorylation of PINK1 by various kinases can modulate its stability and activity, influencing the initiation of mitophagy (11). Similarly, the ubiquitination of mitochondrial proteins serves as a signal for their degradation and is a key step in the mitophagy process (12).

### 2.2 Mitophagy in tumor cell physiology

Mitophagy plays a critical role in the physiology of tumor cells, influencing their survival, metabolic processes, and interactions with the TME (1). Previous studies found that the impact of mitophagy on tumor cells may be bidirectional.

#### 2.2.1 The impact of mitophagy on tumor cell survival

Studies have shown that mitophagy helps eliminate damaged mitochondria, thereby preventing the accumulation of reactive oxygen species (ROS) and maintaining mitochondrial function, which is crucial for cell survival (13). In tumor cells, this process is often upregulated to counteract the detrimental effects of hypoxia and nutrient deprivation, conditions commonly found within solid tumors. For instance, hypoxic tumor cells utilize mitophagy to facilitate metabolic adaptation and mitochondrial renewal, which enhances their survival under stress conditions (14). Moreover, the inhibition of mitophagy has been linked to increased apoptosis in cancer cells. For example, azithromycin, a macrolide antibiotic, has been shown to inhibit mitophagy in hypoxic lung cancer cells, leading to impaired removal of damaged mitochondria and subsequent cell death (15).

Conversely, excessive mitophagy triggers catastrophic mitochondrial loss, irreversibly collapsing energy metabolism by depleting ATP-generating organelles and disabling OXPHOS complexes. This bioenergetic crisis induces ferroptosis via iron-mediated lipid peroxidation and caspase-independent apoptosis through AIF release, culminating in tumor cell death even without therapeutic intervention (16).

#### 2.2.2 The connection between mitophagy and tumor cell metabolism

Mitophagy is intricately linked to the metabolic reprogramming that occurs in cancer cells. Cancer cells often shift their metabolism from oxidative phosphorylation to glycolysis, a phenomenon known as the Warburg effect. However, even in glycolytic tumors, mitochondria remain essential for various metabolic processes, including the generation of ATP and the regulation of metabolic intermediates (17). Research has demonstrated that the activation of mitophagy can enhance mitochondrial metabolism, particularly under conditions of nutrient deprivation. For instance, during periods of glucose starvation, selective autophagy has been shown to activate cyclic AMP protein kinase A (PKA), which in turn rejuvenates mitochondrial function and promotes ATP production. This metabolic adaptation is crucial for tumor cells to thrive in hostile environments, where nutrient availability is often limited (18). Furthermore, mitophagy is involved in the regulation of key metabolic pathways, including fatty acid oxidation and amino acid metabolism (14). The interplay between mitophagy and metabolic pathways highlights its significance in supporting the bioenergetic demands of rapidly proliferating tumor cells.

#### 2.2.3 Interactions between mitophagy and the tumor microenvironment

TME is a complex milieu that significantly influences tumor progression and response to therapy (19). Mitophagy not only affects tumor cell metabolism and survival but also plays a crucial role in modulating TME. For instance, cancer-associated fibroblasts (CAFs) can provide bioavailable iron to tumor cells, promoting resistance to autophagy inhibition (20). Moreover, mitophagy has been implicated in the communication between tumor cells and immune cells within the TME. Autophagy can modulate the release of extracellular vesicles (EVs) containing mitochondrial RNAs, which may influence immune responses and tumor progression (17).

The ability of tumor cells to adapt their mitochondrial function through autophagy allows them to thrive in the immunosuppressive environment typical of many tumors, further complicating treatment strategies. In glioblastoma, for example, NIX-mediated mitophagy has been shown to regulate tumor survival in hypoxic conditions, highlighting the importance of mitophagy in the context of the TME (21). The activation of mitophagy in response to hypoxia not only supports tumor cell survival but also affects the behavior of surrounding immune cells. Disturbed mitochondrial dynamics promoted CD8^+^ T cell exhaustion (22). And mitophagy affected the tumor-associated macrophages, thereby promoting breast cancer progression (4).

### 2.3 Mitophagy in the treatment of solid tumors

#### 2.3.1 The impacts on chemotherapy, radiotherapy, and immunotherapy

**FIGURE 1:**
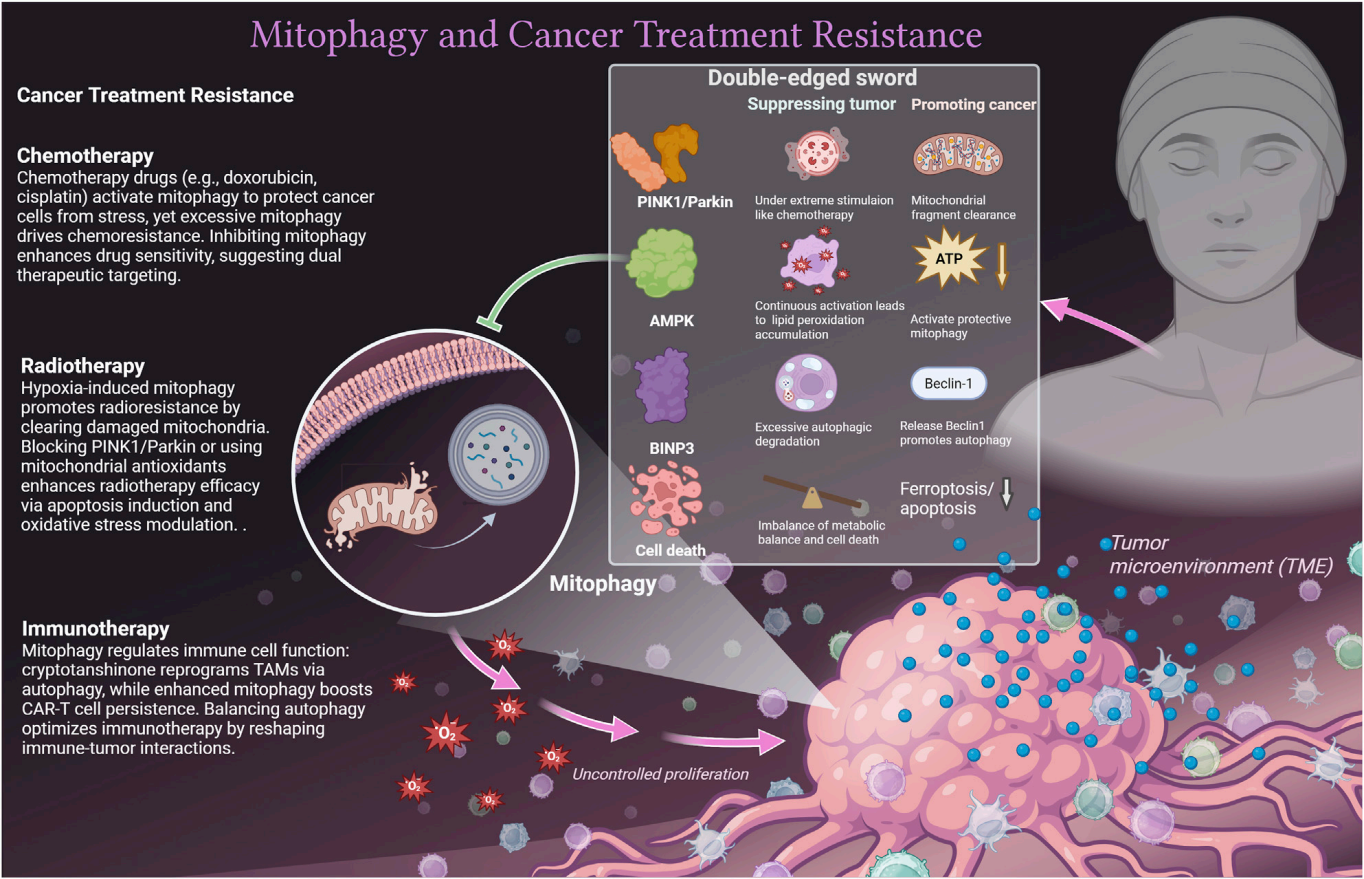
The unique role of mitophagy in different cancer treatment methods with dual effects.

Mitophagy exhibits complex roles in chemotherapy, radiotherapy, and immunotherapy, modulated by TME, immune dynamics, and biological signal networks (23). Mitophagy also interacts with other cell death pathways, such as ferroptosis (24, 25) and apoptosis, which further redirects its role in tumor cells. Due to dynamic changes in TME, the same molecule may play a dual role in mitophagy in tumor cells. For instance, on one side, the AMPK activation in colorectal cancer inhibits tumor growth through Parkin-dependent mitophagy (26). On the other side, under nutritional deprivation or chemotherapy stress, AMPK-activated mitophagy helps cancer cells clear dysfunctional mitochondria and maintain survival, such as in lung cancer cells (27). Similarly, in chronic moderate oxidative stress, Parkin may maintain tumor cell survival by mediating mitochondrial fragment clearance after phosphorylation (28). Under the strong mitochondrial damage stimulation of chemotherapy, the overactivation of the PINK1/Parkin pathway can lead to fatal autophagic lysosome rupture, resulting in tumor cell death (29). Another case is BNIP3, which can competitively bind Bcl-2 and release Beclin1 to promote autophagy and protect tumor cells (30). Under extreme stimulation, it can form a complex with BNIP3L to induce excessive autophagic degradation in tumor cells (31). We emphasize the unique role of mitophagy in different cancer treatment methods and explain the double-edged sword mechanism (Figure 1).

Mitophagy exerts dual effects across cancer therapies: In chemotherapy, doxorubicin-induced mitophagy confers cellular protection (24) but exacerbates resistance when amplified (23). Proteasome inhibitor ONX0912 activates Parkin/PINK1-mediated mitophagy to trigger apoptosis in liver cancer cells (32). Mitophagy inhibition enhances cisplatin sensitivity and reduces toxicity (33), and metformin exploits mitophagy for chemosensitization and pro-apoptotic effects (2). Regarding radiotherapy, tumor hypoxia initiates pro-survival mitophagy, driving radioresistance (34), whereas blocking the PINK1/Parkin axis (35) or administering mitochondrial-targeted antioxidants (36) restores radiosensitivity. For immunotherapy, cryptotanshinone repolarizes TAMs toward an antitumor M1 phenotype via suppressed oxidative phosphorylation and autophagy induction (37), with nanovaccines augmenting TAM-mediated immunoresponse (38). Recent studies showed that enhancing mitophagy in CAR-T cells bolsters their persistence and functionality in the TME (39), though uncontrolled suppression compromises effector immunity (40), necessitating balanced modulation strategies.

#### 2.3.2 Cancer cell-specific resistance mechanisms

The cancer cells exhibit a range of resistance mechanisms that enable them to survive therapeutic interventions, with mitophagy playing a pivotal role in this process. In the context of cisplatin resistance in osteosarcoma, the FoxG1/BNIP3 axis has been implicated in regulating mitophagy. Studies have shown that cisplatin-resistant cells exhibit downregulation of FoxG1 and BNIP3, leading to impaired mitophagy (3). Overexpression of FoxG1 was found to enhance BNIP3 expression and restore mitophagic activity, thereby resensitizing resistant cells to cisplatin treatment. This highlights the potential of targeting specific mitophagy-related pathways to overcome drug resistance in cancer therapy. In hepatocellular carcinoma (HCC), the interplay between mitophagy and drug resistance has also been explored. Research indicates that hyperactivated mitophagy, regulated by the ATAD3A-PINK1/PARKIN axis, is essential for sorafenib resistance in HCC cells. Inhibition of ATAD3A was shown to restore sensitivity to sorafenib by disrupting the mitophagic process, suggesting that targeting this pathway could provide a novel therapeutic strategy for patients with HCC who exhibit resistance to standard treatments (41). Furthermore, the role of mitophagy in mediating resistance to doxorubicin in breast cancer has been investigated, with evidence suggesting that increased mitophagic flux correlates with resistance to this chemotherapeutic agent. The expression of miR-218-5p, which targets Parkin, was shown to inhibit doxorubicin-induced mitophagy, thereby enhancing the sensitivity of resistant breast cancer cells to treatment (42). This underscores the potential of utilizing miRNA-based therapies to modulate mitophagy and enhance the efficacy of existing chemotherapeutics.

### 2.4 Research directions to improve mitophagy-related tumor treatment

#### 2.4.1 Targeting mitophagy therapies

Mitophagy critically maintains cellular homeostasis and influences cancer resistance by regulating mitochondrial quality control, enabling cells to eliminate damaged organelles and thereby reducing oxidative stress. Recent evidence highlights its therapeutic potential, but its dual role and the heterogeneity between different tumor types pose challenges and difficulties for direct intervention. Future research may need to first balance the dual role and interactive effects of mitophagy itself on target tumor cells. Combining the dual effects of mitophagy molecules mentioned earlier, it suggests that clinical AMPK inhibitors should be used before radiotherapy (to block protective autophagy). And PINK1 activators need to be administered 24 h after chemotherapy (peak period of promoting death).

Meanwhile, researchers can consider multiple targets to overcome the adverse effects of TME on targeted mitophagy. For example, HIF1α inhibitors may overcome hypoxia-induced resistance by blocking BNIP3L-mediated mitophagy (3). New techniques like engineering tumor-targeted nanoparticles can deliver metal complexes (e.g., copper-phenanthroline) that specifically disrupt PARK2-dependent lysosomal clearance (43). Intravital imaging probes can visually quantify mitophagy flux during chemotherapy penetration in spatially resolved tumor niches, enabling real-time optimization of combinatorial scheduling against adaptive resistance mechanisms.

#### 2.4.2 Potential of combination therapy strategies

Combination therapy strategies have emerged as a promising avenue in overcoming resistance. We identified drug targets from the perspective of mitophagy and listed corresponding drugs and potential mechanisms of action (Table 1). Table 1 includes targets that promote mitophagy to enhance solid tumor treatment, such as BNIP3/NIX. Listed content also includes strategies to inhibit excessive mitochondrial fission (such as DRP1 inhibitor (44)) to weaken the redox homeostasis of tumor cells and increase their sensitivity to radiotherapy and chemotherapy.

**TABLE 1:** Mitophagy targets and drug development.

Target	Compound	Mechanism	Cancer types	Stage
BNIP3/NIX	NecroX-5	Activates BNIP3-dependent mitophagy	Breast cancer	Preclinical (45)
PINK1/Parkin	Urolithin A	Enhances PINK1/Parkin-mediated clearance	Prostate cancer, Glioblastoma	Preclinical/Phase I (46)
HSP90	Geldanamycin	Disrupts HSP90-p53 interaction, induces mitophagy	Lung cancer, Breast cancer	Preclinical (47, 48)
DRP1	Mdivi-1	Disrupts the redox homeostasis maintained by fission-mitophagy	Breast cancer, Thyroid cancer	Preclinical (49, 50)
P62/SQSTM1	Metformin	Activates P62-dependent mitophagy	Breast cancer, Prostate cancer, Ovarian cancer	Clinical trials (51–53) (repurposed)
ULK1	SBI-0206965	Activates ULK1-mediated mitophagy	Glioblastoma, Lung c cancer	Preclinical (54, 55)
FUNDC1	Artesunate	Triggers FUNDC1-dependent mitophagy	Hepatocellular carcinoma	Preclinical (56)
BCL-2/BCL-xL	Venetoclax	Synergizes with mitophagy inhibition	Leukemia	Approved (combined) (57)
mTOR	Rapamycin (Sirolimus)	Inhibits mTOR, enhances mitophagy	Renal cell carcinoma, Breast cancer	Approved (repurposed) (58)
NIX/BNIP3L	Cisplatin	Chemotherapy-induced mitophagy	Multiple cancers	Approved (adjuvant) (3)

Recent studies have also explored the use of combination therapies that incorporate novel agents targeting specific cellular mechanisms, such as mitophagy (59). By concurrently inhibiting mitophagy while administering traditional chemotherapeutics, researchers have observed a significant increase in cancer cell sensitivity to treatment. Furthermore, the development of targeted radionuclide therapies in conjunction with chemotherapeutics has shown promise in enhancing treatment efficacy while minimizing off-target effects. However, it is worth noting that most drugs targeting mitophagy targets are still in the preclinical research stage. This may be due to a lack of isoform specificity for the target protein, like PINK1 (2), which may increase the potential of off-target effects. Heterogeneity in mitophagy receptors (e.g., FUNDC1 (56) and BNIP3 (3)) across cancer types creates inconsistent therapeutic responses. And systemic inhibition of mitophagy disrupts mitochondrial quality control in vital organs (e.g., neurons, cardiomyocytes), triggering off-target toxicity like neurodegeneration or cardiac dysfunction.

Future research may focus on identifying optimal drug combinations tailored to individual patient profiles. Additionally, the exploration of biomarkers that predict response to combination therapies could facilitate more personalized treatment approaches (60). One study demonstrated that mitophagy-related genes can be prognostic biomarkers and therapeutic targets of gastric carcinoma (61). Moreover, combining materials science to design drug-targeted mitophagy and other related pathways may be a new direction for solid tumor treatment t (16, 25, 62, 63).

## 3 Conclusion

Mitophagy emerges as a critical mechanism driving treatment resistance in solid tumors. This review synthesizes evidence demonstrating that cancer cells exploit mitophagy to survive therapeutic stress (chemotherapy, radiotherapy, immunotherapy) and harsh microenvironments (hypoxia, nutrient deprivation). Mitophagy promotes resistance by clearing damaged mitochondria, maintaining metabolic fitness, reducing oxidative stress, suppressing ferroptosis, and facilitating immune evasion. Key regulators like PINK1/Parkin and BNIP3/FUNDC1 are frequently implicated. While inhibiting aberrant mitophagy can sensitize tumors to treatment (e.g., restoring cisplatin/sorafenib sensitivity), systemic inhibition risks disrupting essential mitochondrial quality control in healthy tissues. Conversely, enhancing mitophagy in specific contexts (e.g., CAR-T cells) may improve immunotherapy efficacy. Current mitophagy-targeting strategies, though promising in preclinical studies, face challenges including target specificity, tumor heterogeneity, and potential off-target toxicity. Future research must focus on developing isoform-specific modulators, identifying predictive biomarkers, and designing rational combination therapies that exploit mitophagy’s dual roles. Understanding the intricate crosstalk between mitophagy, tumor metabolism, and the microenvironment remains paramount for translating these insights into effective clinical strategies to overcome treatment resistance.
